# Knowledge, attitudes, and preventive behaviors toward rabies among urban residents in Bangkok, Thailand: Implications for targeted One Health rabies elimination strategies

**DOI:** 10.14202/vetworld.2026.4159-4174

**Published:** 2026-09-22

**Authors:** Kittithat Luenglertamorn, Taksaporn Borvornpadungkitti, Rapee Luangyawee, Siwat Choksuchat, Atithan Paoya, Anisa Techakunpongkorn, Seema Vijay Medhe, Manoj Tukaram Kamble, Saharuetai Jeamsripong

**Affiliations:** 1 Sixth-year Veterinary Student, Faculty of Veterinary Science, Chulalongkorn University, Bangkok 10330, Thailand.; 2 ASEAN Institute for Health Development, Mahidol University, Salaya, Phutthamonthon 73170, Nakhon Pathom, Thailand.; 3 Department of Agricultural and Fishery Science, Faculty of Science and Technology, Prince of Songkla University, Pattani 94000, Thailand.; 4 Department of Veterinary Public Health, Faculty of Veterinary Science, Chulalongkorn University, Bangkok 10330, Thailand.; 5 Research Unit in Microbial Food Safety and Antimicrobial Resistance, Department of Veterinary Public Health, Faculty of Veterinary Science, Chulalongkorn University, Bangkok 10330, Thailand.

**Keywords:** Bangkok, knowledge, One Health, preventive behaviors, rabies, public awareness, Thailand, urban rabies control

## Abstract

**Background and Aim:**

Rabies remains a fatal zoonotic disease and an important public health concern despite substantial reductions in human cases in Thailand. Continued circulation of rabies among animal populations highlights the need to strengthen public awareness and preventive behaviors. This study aimed to assess knowledge, attitudes, and preventive behaviors regarding rabies among Bangkok residents, identify sociodemographic factors associated with these outcomes, and examine relationships among knowledge, attitudes, and preventive behaviors to support urban rabies prevention and control strategies.

**Materials and Methods:**

A cross-sectional online survey was conducted among Bangkok residents between December 2024 and August 2025 using a structured, content-validated questionnaire. Of 262 respondents, 258 met the eligibility criteria and were included in the analysis. Descriptive statistics summarized participant characteristics and knowledge, attitudes, and preventive behaviors. Binary logistic regression identified factors associated with these domains, while Pearson’s chi-square test evaluated associations among knowledge, attitudes, and preventive behaviors.

**Results:**

Overall, 58.9% of participants demonstrated sufficient knowledge, whereas attitudes and preventive behaviors were generally favorable. Correct responses were highest for rabies transmission (97.7%), clinical signs in dogs (98.8%), and animal vaccination (96.9%), whereas only 36.1% correctly identified the recommended observation period following a bite incident. Participation in rabies awareness activities was limited (26.7%), although 73.6% reported routine pet rabies vaccination. Multivariable logistic regression showed that private sector employees were less likely to demonstrate sufficient knowledge (adjusted odds ratio [AOR] = 0.268, 95% confidence interval [CI]: 0.148-0.483; p < 0.001) and positive attitudes (AOR = 0.337, 95% CI: 0.186-0.610; p < 0.001) than students/unemployed participants. Participants aged 26-40 years had lower odds of appropriate preventive behaviors (AOR = 0.338, 95% CI: 0.173-0.657; p = 0.001), whereas participants whose pets had received rabies vaccination were more likely to report appropriate preventive behaviors (AOR = 2.512, 95% CI: 1.413-4.465; p = 0.002). Significant positive associations were identified between knowledge and attitudes and between knowledge and preventive behaviors (both p < 0.001).

**Conclusion:**

Although participants generally demonstrated favorable rabies-related knowledge, attitudes, and preventive behaviors, important gaps persisted regarding post-bite animal observation and participation in rabies awareness activities. Occupation and age were important determinants of knowledge, attitudes, and preventive behaviors, while responsible pet vaccination was positively associated with preventive practices. These findings support targeted, culturally appropriate educational interventions, workplace- and community-based awareness programs, and stronger One Health collaboration to advance urban rabies prevention and support Thailand’s progress toward the WHO “Zero by 30” rabies elimination goal.

## INTRODUCTION

Rabies is a viral zoonotic disease that affects the central nervous system of mammals, resulting in severe neurological manifestations and, once clinical signs appear, almost inevitable death [1, 2]. In dogs and cats, the clinical course of rabies is commonly divided into three stages: The prodromal stage, the furious (excitatory) stage, and the paralytic (dumb) stage. Recognizing these stages is essential for early diagnosis, timely case management, and prompt implementation of public health interventions to reduce transmission risk [3, 4].

Rabies is primarily transmitted to humans through bites from infected animals [5, 6]. Following inoculation, the virus initially replicates in muscle tissue at the bite site before entering peripheral nerves and migrating to the spinal cord and brain. After the virus establishes infection in the central nervous system, it spreads centrifugally to the salivary glands, where it is shed in saliva, facilitating transmission through biting [7, 8]. Although bites represent the principal route of exposure, rabies virus infection may also occur through contamination of open wounds or mucous membranes with infectious saliva [5].

Rabies is among the deadliest infectious diseases, with a case-fatality rate approaching 100% once clinical signs develop [9, 10]. In Thailand, human rabies incidence has declined substantially over the past four decades, whereas reported animal rabies cases have increased over the last decade. Rabies was officially designated a notifiable disease in Thailand in 1980, when 370 human cases were reported. This number subsequently declined to 185, 50, 15, and 2 cases in 1990, 2000, 2010, and 2020, respectively [11, 12]. Despite this marked reduction in human cases, rabies remains endemic in animal populations. Using national surveillance data from the Department of Livestock Development, Punyapornwithaya *et al.* [11] projected approximately 278, 288, and 300 confirmed canine rabies cases in 2023, 2024, and 2025, respectively, indicating continued viral circulation and highlighting the need for sustained surveillance, vaccination, and public awareness programs. Vaccination remains the cornerstone of rabies prevention because it is safe, highly effective, and cost-effective for interrupting transmission [6, 13–16]. Additional control measures include responsible pet ownership, management of free-roaming animal populations, public education, environmental sanitation, and, where appropriate, oral vaccination of wildlife reservoirs.

Knowledge, attitudes, and practices (KAP) surveys are widely used to evaluate what individuals know, believe, and do regarding specific health issues. They provide a practical framework for assessing health-related behaviors [17], identifying misconceptions and behavioral gaps, and establishing baseline information to guide the development and evaluation of public health interventions [18]. In rabies control programs, KAP studies help veterinarians, physicians, and public health authorities identify priority populations for educational interventions, improve community awareness, and strengthen disease prevention strategies.

Rabies remains a major global public health concern, particularly in Asia and Africa [19]. The World Health Organization (WHO), together with the Food and Agriculture Organization, the World Organization for Animal Health, and the United Nations Children's Fund (UNICEF), has established the global "Zero by 30" initiative to eliminate dog-mediated human rabies by 2030 [20, 21]. Achieving this target requires sustained public awareness, community engagement, and evidence-based prevention strategies. However, Thailand still has important knowledge and behavioral gaps. Previous studies have reported inadequate rabies knowledge among more than half of schoolchildren, limited awareness of non-canine rabies reservoirs, insufficient understanding of appropriate wound management following animal bites, and socioeconomic barriers affecting vaccine uptake and willingness-to-pay among dog owners [22–24].

Bangkok, the capital city of Thailand, presents distinctive challenges for rabies prevention because of its high human population density, extensive pet ownership, and frequent interactions between humans and free-roaming animals. The Greater Bangkok area is estimated to have approximately 1.3 million owned dogs, of which 66% are allowed to roam freely, and 46% are acquired directly from the street, increasing opportunities for rabies transmission [25, 26]. Despite these risk factors, a recent survey reported that only 17.4% of workers in the Bangkok metropolitan area demonstrated good knowledge of rabies [27]. Most previous KAP studies in Thailand have focused on specific groups, including schoolchildren, dog owners, or occupational populations, whereas contemporary evidence describing rabies-related KAP among the general adult population in Bangkok remains limited. Furthermore, few studies have simultaneously examined the influence of sociodemographic characteristics on KAP outcomes and the interrelationships among KAP within a highly urbanized setting. Addressing these gaps is essential for developing targeted educational interventions and urban rabies control policies that support Thailand's contribution to the WHO "Zero by 30" initiative. Findings from Bangkok may also provide valuable evidence for other rapidly urbanizing cities in Southeast Asia facing similar challenges in rabies prevention and control.

This study aimed to assess the KAP regarding rabies among adult residents of Bangkok, Thailand. Specifically, the study sought to (i) evaluate the level of rabies-related KAP among participants, (ii) identify sociodemographic factors associated with KAP outcomes using multivariable analysis, and (iii) examine the relationships among KAP. The findings are expected to provide evidence to support targeted educational interventions, strengthen urban rabies prevention and control programs, and inform One Health strategies to achieve the WHO "Zero by 30" rabies elimination goal.

## MATERIALS AND METHODS

### Ethical approval

The study protocol was reviewed and approved by the Research Ethics Review Committee for Research Involving Human Participants, Group I, Chulalongkorn University, Thailand (Certificate of Approval No. 090/68, 16-04-2025). Ethical approval was obtained before participant recruitment and data collection. All participants were aged ≥18 years and provided electronic informed consent before accessing and completing the online questionnaire. Participants were informed of the study objectives, procedures, voluntary nature of participation, and their right to withdraw before submitting their responses. No personally identifying information was collected through the questionnaire, and responses were handled confidentially and analyzed in aggregate to protect participant privacy. The study was conducted in accordance with the approved research protocol and applicable ethical principles for research involving human participants.

### Study period and location

This cross-sectional study was conducted between April 2025 and August 2025 among adult residents of Bangkok, Thailand. Participant recruitment used a mixed-channel online strategy that combined social media platforms (Facebook, Instagram, and LINE) with quick response (QR) code posters displayed at public locations throughout Bangkok. This recruitment approach was selected to maximize participation from geographically diverse urban residents and improve accessibility through widely used digital communication platforms.

### Study design

A questionnaire-based cross-sectional survey was designed to evaluate KAP regarding rabies among residents of Bangkok, Thailand. The study also investigated sociodemographic factors associated with KAP outcomes and examined the relationships among KAP.

### Sample size calculation

The required sample size was calculated using Cochran's formula, which is widely applied in cross-sectional studies involving large populations [28]:


\[
n=\frac{P\left(1-P\right){Z}^{2}}{{d}^{2}}
\]

where *n* represents the required sample size, *P* is the expected prevalence (assumed response rate of 80%), *Z* is the standard normal deviate corresponding to a 95% confidence interval (CI; 1.96), and *d* is the desired precision (0.05).

Based on this calculation, the minimum required sample size was 246 participants. A total of 262 individuals completed the survey; 258 met the eligibility criteria and were included in the final analysis.

### Participants

Participants were recruited using a non-probability convenience sampling approach. The survey link was disseminated through Facebook, Instagram, and LINE, together with QR-code posters displayed at public locations across Bangkok. Participation was voluntary, and eligible individuals self-selected to complete the anonymous online questionnaire.

Eligible participants were required to be ≥18 years of age, reside in Bangkok, have regular contact with animals through pet ownership, environmental exposure, or residence in communities where rabies posed a potential risk, and be able to read and understand the Thai language because the questionnaire was administered in Thai.

Individuals were excluded if they were <18 years of age, were not residents of Bangkok, were unable to provide informed consent, or submitted incomplete questionnaires. Educational attainment was not used as an exclusion criterion.

### KAP questionnaire

The questionnaire consisted of four sections.

The first section collected participants' demographic information, including sex, age, education level, occupation, and pet ownership.

The second section evaluated rabies knowledge using 10 questions designed to assess participants' understanding of the disease. Most questions were multiple-choice, while one question (K7) allowed multiple correct responses, such as the causes of rabies and modes of rabies transmission.

The third section assessed attitudes toward rabies using 10 statements rated on a 5-point Likert scale ranging from 1 (strongly disagree) to 5 (strongly agree). Representative statements included the perceived importance of vaccinating dogs against rabies.

The fourth section evaluated rabies-related practices using seven questions addressing preventive behaviors. Three questions (P2, P3, and P5) included multiple independently scored preventive behaviors, whereas the remaining questions each represented a single behavioral item. Consequently, the practice domain consisted of 14 independently scored behavioral items, resulting in a maximum possible score of 14. Each appropriate preventive behavior received one point, whereas inappropriate behaviors received zero points according to the Thai Department of Livestock Development rabies prevention guidelines.

Representative questionnaire items are presented in the main text, whereas the complete questionnaire is provided in Supplementary Questionnaire S1.

### Questionnaire validation

The questionnaire was developed based on previously published rabies KAP studies and adapted to the Thai context according to the Rabies Clinical Practice Guidelines issued by the Thai Department of Livestock Development. The questionnaire structure and validation procedures were also informed by previously published questionnaire-based studies conducted in Thailand [29].

Content validity was assessed using the Item-Objective Congruence (IOC) index by eight experts in veterinary public health, veterinary medicine, and research methodology, each with more than 8 years of professional experience. The overall IOC score was 0.80, indicating acceptable content validity.

Before the main survey, the questionnaire was pilot-tested among 10 participants representative of the target population to evaluate clarity, comprehensibility, and feasibility. Feedback from the pilot study was incorporated to improve wording and readability before administering the final questionnaire. Internal consistency of the attitude section was assessed using Cronbach's alpha, which yielded a coefficient of 0.741, indicating acceptable reliability.

### Data collection

The questionnaire was administered online using Google Forms (Google LLC, Mountain View, CA, USA). The survey link was distributed through Facebook, Instagram, and LINE, together with posters containing QR codes displayed at public locations throughout Bangkok. Google Forms enabled standardized electronic data collection, secure data management, real-time data export, and reduced transcription errors.

To minimize duplicate responses, Google Forms was configured to accept only one submission per Google account. The dataset was subsequently screened for duplicate entries before statistical analysis.

Raw data were exported to Microsoft Excel (Microsoft Corporation, Redmond, WA, USA) for data management and scoring.

Knowledge scores were calculated by assigning one point for each correct response and zero points for incorrect or unanswered responses.

Attitude and practice responses were evaluated according to the Rabies Clinical Practice Guidelines issued by the Thai Department of Livestock Development. Appropriate responses received one point, whereas inappropriate responses received zero points. For the practice domain, each independently scored appropriate preventive behavior received one point.

Scores for each domain were converted into percentages. Participants achieving scores ≥80% were classified as having sufficient KAP (coded as 1), whereas those scoring <80% were classified as having insufficient KAP (coded as 0). The ≥80% threshold was based on the modified Bloom's cutoff, widely used in KAP studies to distinguish adequate from inadequate KAP [30, 31].

The complete questionnaire is provided in Supplementary Questionnaire S1.

### Statistical analysis

Descriptive statistics were used to summarize participant characteristics and KAP outcomes, including frequencies, percentages, means, and standard deviations.

Binary logistic regression analyses were performed to identify factors associated with KAP regarding rabies. Independent variables included sex, age, education level, occupation, pet ownership, and pet vaccination history. Pet vaccination history was included because responsible pet vaccination may reflect preventive health behavior and engagement with veterinary services, which could influence rabies-related knowledge and preventive practices.

Variables with p < 0.20 in the univariable logistic regression analyses, together with epidemiologically relevant variables, were entered into multivariable logistic regression models to adjust for potential confounding. Reference categories were selected according to epidemiological relevance and sample size, with the largest category serving as the reference group where appropriate. Adjusted odds ratios (AORs) with 95% CIs were calculated. Statistical significance in the multivariable analyses was defined as p < 0.05.

Only complete questionnaires were included in the final analysis; therefore, no missing data were present and no imputation procedures were required. Multicollinearity among independent variables was assessed using variance inflation factors, and no evidence of problematic multicollinearity was identified.

All statistical analyses were performed using IBM SPSS Statistics version 30 (IBM Corp., Armonk, NY, USA).

## RESULTS

### Sociodemographic characteristics of the participants

A total of 262 individuals participated in the survey. Of these, 258 respondents met the inclusion criteria and were included in the final analysis, yielding a questionnaire completion rate of 98.47% (258/262). Participants were recruited from multiple districts across Bangkok (Figure 1), providing broad geographic representation of the metropolitan area and supporting the interpretation of the findings within an urban public health context.

**Figure 1:**
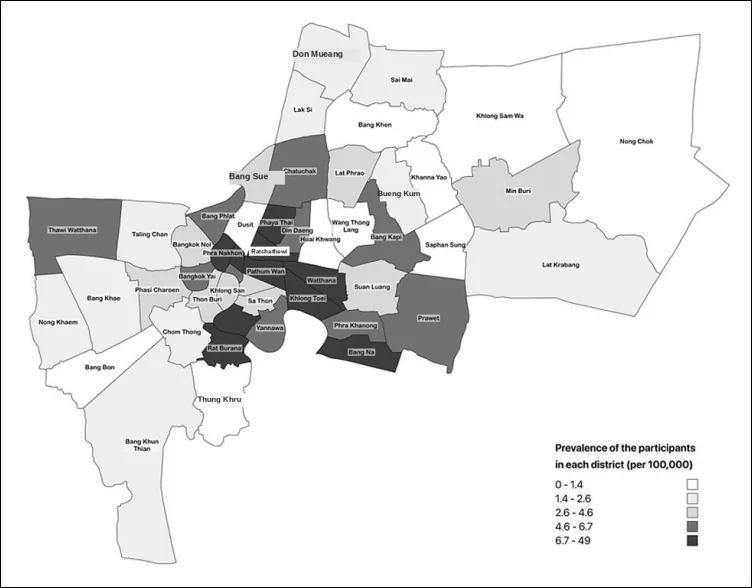
Distribution of participants by district of residence in Bangkok (n = 258).

The majority of respondents were female (70.93%), and the largest age group was 18-25 years (51.55%). Nearly half of the participants were students or unemployed (48.06%), and most held a bachelor's degree (72.48%). Regarding pet ownership, 64.70% of respondents reported owning one or more pets. Overall, 60.47% of all participants reported that their pets had received rabies vaccination. Table 1 presents the detailed sociodemographic characteristics of the participants.

**Table 1:** Sociodemographic characteristics of study participants in Bangkok (n = 258).

**Variable**	**n (%)**
Gender	
Male	69 (26.7)
Female	183 (70.9)
Not specified	6 (2.3)
Pet ownership	
None	91 (35.3)
Has pet(s)	167 (64.7)
Age (years)	
18–25	133 (51.6)
26–40	57 (22.1)
41–60	55 (21.3)
>60	13 (5.0)
Educational level	
Vocational/high vocational certificate/secondary school	27 (10.5)
Bachelor’s degree	187 (72.5)
Master’s degree	44 (17.1)
Occupation	
Student/unemployed	124 (48.1)
Public sector	16 (6.2)
Private sector	82 (31.8)
Self-employed/entrepreneur	32 (12.4)
Healthcare professional (e.g., physiotherapist, nurse, veterinarian)	4 (1.6)
Pet vaccination status	
Never received/no pets	86 (33.3)
No pets requiring vaccination	16 (6.2)
Has received	156 (60.5)

Values are presented as number (%). Totals may not equal 100% because of rounding or missing responses.

### Rabies knowledge

Participants' knowledge regarding rabies was assessed using 10 multiple-choice questions, with total scores ranging from 0 to 10. The mean knowledge score was 7.75. Based on the predefined cutoff, 58.9% of participants demonstrated sufficient knowledge by correctly answering at least 80% of the questions. The distribution of correct responses for each knowledge item is presented in Figure 2.

**Figure 2:**
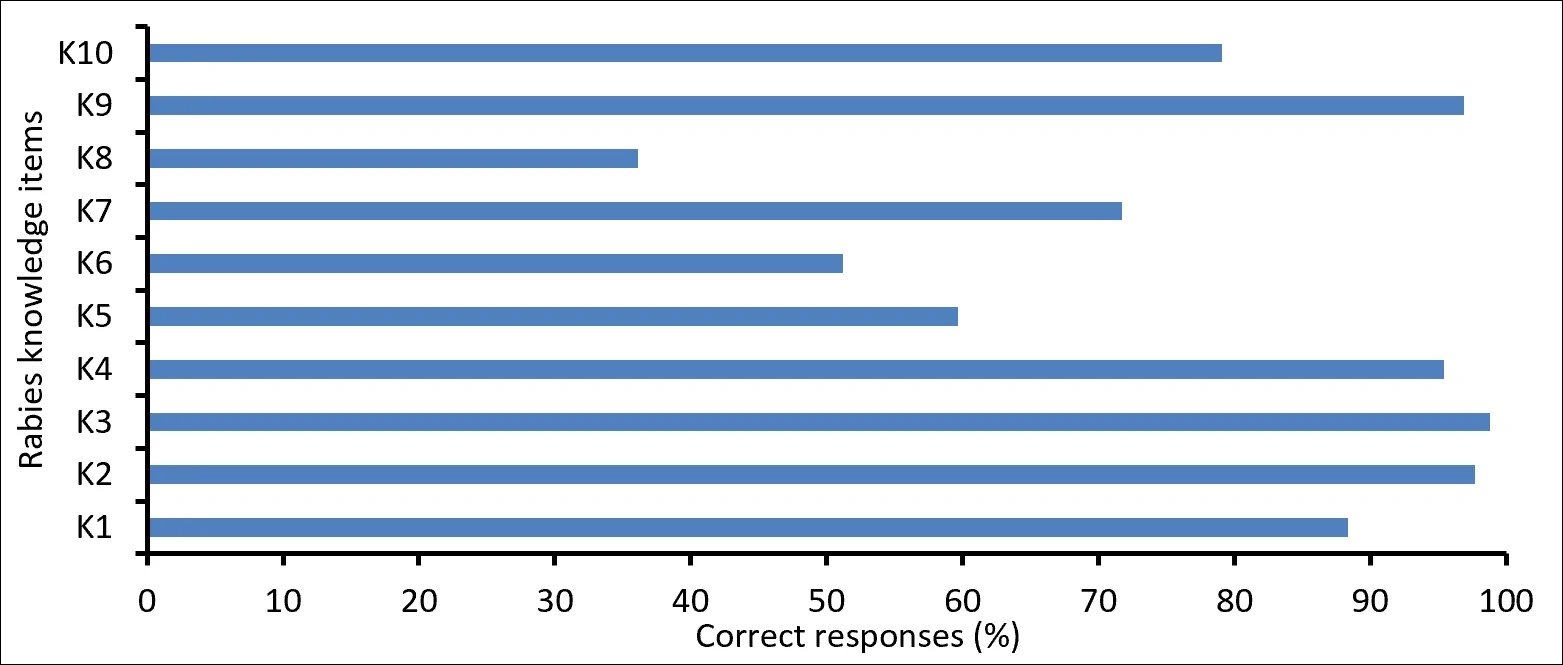
Percentage of correct responses to individual rabies knowledge items among participants (n = 258). K1-K10 represent the knowledge domains assessed in the questionnaire. Responses were classified as correct or incorrect according to standard rabies prevention guidelines. K1 = Cause of rabies; K2 = Transmission routes; K3 = Clinical signs in dogs; K4 = Susceptible animal species; K5 = Human rabies curability; K6 = Age for first puppy vaccination; K7 = Reporting authorities; K8 = Post-bite observation period; K9 = Animal vaccination practices; K10 = Seasonal occurrence.

The highest proportions of correct responses were observed for questions related to rabies transmission (K2, 97.7%), clinical signs in dogs (K3, 98.8%), and rabies vaccination in animals (K9, 96.9%). In contrast, the lowest proportion of correct responses was recorded for K8, which assessed the recommended observation period following a bite incident, with only 36.1% of participants answering correctly.

### Attitudes toward rabies

Attitudes toward rabies prevention and control were assessed using 10 Likert scale statements, with total scores ranging from 0 to 10. The mean attitude score was 7.72, indicating generally favorable attitudes toward rabies prevention. More than 85% of participants demonstrated appropriate attitudes for five of the 10 statements (Figure 3).

**Figure 3:**
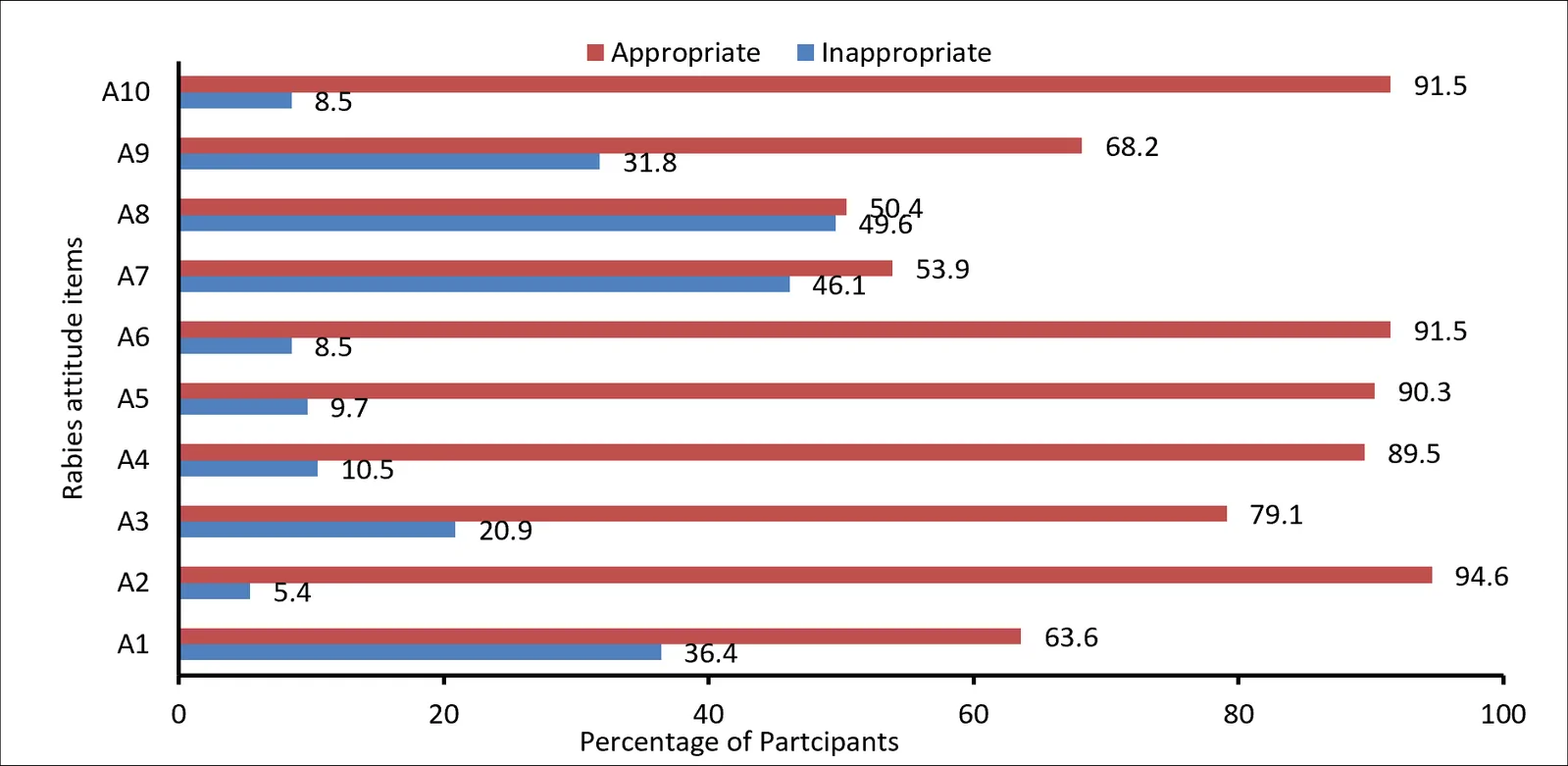
Percentage of participants providing appropriate and inappropriate responses to rabies attitude statements (n = 258). Responses were classified as appropriate or inappropriate according to the Thai Department of Livestock Development rabies prevention guidelines. Exact percentages are displayed on each bar. A1 = Rabies is no longer a concern; A2 = Importance of dog vaccination; A3 = Booster vaccination is unnecessary after a dog bite; A4 = Confinement of a suspected rabid dog; A5 = Reporting a suspected rabid dog to the appropriate authorities; A6 = Vaccination of stray animals; A7 = Euthanasia of confirmed rabid dogs; A8 = Ability to recognize a rabid dog; A9 = Human rabies vaccination; A10 = Rabies awareness reduces the risk of infection.

The highest proportion of appropriate responses was observed for A2, which assessed the importance of vaccinating dogs against rabies (94.6%). In contrast, attitudes regarding euthanasia of confirmed rabid dogs (A7) and participants' confidence in recognizing rabies in dogs (A8) were more variable, with appropriate responses reported by 53.9% and 50.4% of participants, respectively.

### Rabies-related practices

Rabies-related practices were evaluated using seven practice-based questions, with total scores ranging from 0 to 14. The mean practice score was 11.05, indicating generally appropriate rabies prevention practices among the participants. Figure 4 presents the distribution of appropriate and inappropriate preventive practices across the seven practice domains.

**Figure 4:**
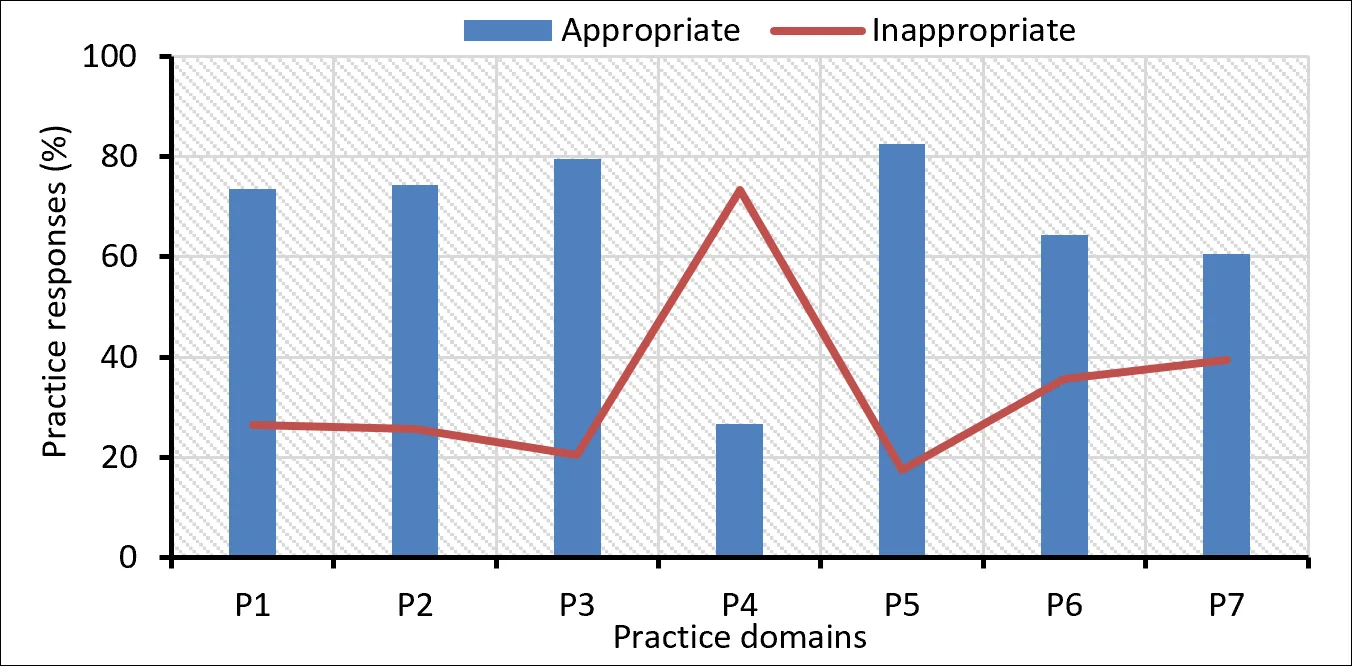
Distribution of appropriate and inappropriate rabies-related preventive practices among participants (n = 258). Each point represents the proportion of respondents reporting appropriate and inappropriate behaviors across seven rabies prevention practice domains (P1-P7). For frequency-based items, responses indicating consistent or routine practice (e.g., always or usually) were considered appropriate. For domains comprising multiple behavioral responses (P2, P3, and P5), values represent the overall proportion of participants reporting appropriate actions within each domain. P1 = Routine pet vaccination; P2 = Response to a suspected rabid stray dog; P3 = Action after a stray dog bite; P4 = Participation in rabies training or awareness campaigns; P5 = Response to a pet dog bite incident; P6 = Sharing rabies-related knowledge with others; P7 = Personal rabies vaccination.

Most participants reported appropriate preventive behaviors. Specifically, 73.6% reported routinely vaccinating their pets against rabies, 95.3% indicated that they would avoid contact with a stray dog showing aggressive behavior and excessive salivation, 92.6% would wash a bite wound immediately with soap and water following a bite from a stray dog of unknown vaccination status, and 95.3% would seek immediate medical attention. In contrast, only 26.7% of participants reported previous participation in rabies-related training or awareness programs, while 60.5% reported having received rabies vaccination themselves.

### Factors associated with rabies knowledge

Univariable logistic regression analysis identified age, educational level, and occupation as factors associated with rabies knowledge (Table S1).

After adjustment for potential confounders, occupation remained the only independent predictor of sufficient rabies knowledge (Table 2). Compared with students or unemployed participants, private sector employees had significantly lower odds of demonstrating sufficient knowledge (AOR = 0.268, 95% CI = 0.148-0.483, p < 0.001). Self-employed participants also had lower odds of sufficient knowledge, although this association was borderline statistically significant (AOR = 0.451, 95% CI = 0.203-1.000, p = 0.050).

### Factors associated with attitudes toward rabies

Univariable analysis demonstrated significant associations between attitudes toward rabies and age, educational level, occupation, and pet ownership (Table S2).

After multivariable adjustment, occupation remained significantly associated with attitudes toward rabies (Table 2). Participants employed in the private sector had significantly lower odds of exhibiting positive attitudes than students or unemployed participants (AOR = 0.337, 95% CI = 0.186-0.610, p < 0.001). No other occupation category remained statistically significant after adjustment.

### Factors associated with rabies-related practices

Univariable logistic regression analysis identified sex, age, occupation, pet ownership, and pet vaccination history as variables associated with rabies-related practices (Table S3).

Multivariable logistic regression demonstrated that age and pet vaccination history were independently associated with preventive practices (Table 2). Compared with participants aged 18-25 years, those aged 26-40 years (AOR = 0.338, 95% CI = 0.173-0.657, p = 0.001), 41-60 years (AOR = 0.366, 95% CI = 0.187-0.715, p = 0.003), and >60 years (AOR = 0.193, 95% CI = 0.050-0.754, p = 0.018) had significantly lower odds of demonstrating appropriate preventive practices. Participants whose pets had received rabies vaccination were more than twice as likely to report appropriate preventive practices than those without vaccinated pets (AOR = 2.512, 95% CI = 1.413-4.465, p = 0.002).

### Multivariable analysis of factors associated with KAP

Multivariable logistic regression analysis was performed to identify independent predictors of KAP regarding rabies (Table 2). The healthcare professional subgroup comprised only four participants; therefore, the corresponding AOR estimates had wide CIs and should be interpreted cautiously.

Occupation was the only independent predictor of both knowledge and attitudes. Private sector employees had significantly lower odds of demonstrating sufficient knowledge and positive attitudes than students or unemployed participants. Regarding preventive practices, increasing age was associated with lower odds of appropriate preventive behaviors, whereas previous pet rabies vaccination was positively associated with appropriate preventive practices.

**Table 2:** Multivariable logistic regression analysis of factors associated with knowledge, attitudes, and practices regarding rabies.

**Variable**	**Insufficient, n (%)**	**Sufficient, n (%)**	**AOR**	**SE**	**95% CI**	**p-value**
**Knowledge**						
Occupation						
Student/unemployed	35 (13.6)	88 (34.2)	Ref.	–	–	–
Public sector	7 (2.7)	9 (3.5)	0.511	0.542	0.177–1.480	0.216
Private sector	49 (19.1)	33 (12.8)	0.268	0.301	0.148–0.483	<0.001*
Self-employed/entrepreneur	15 (5.8)	17 (6.6)	0.451	0.407	0.203–1.000	0.050*
Healthcare professionals	1 (0.4)	3 (1.2)	1.193	1.172	0.120–11.863	0.880
**Attitudes**						
Occupation						
Student/unemployed	31 (12.1)	92 (35.8)	Ref.	–	–	–
Public sector	5 (2.0)	11 (4.3)	0.741	0.578	0.239–2.301	0.605
Private sector	41 (15.6)	41 (15.6)	0.337	0.303	0.186–0.610	<0.001*
Self-employed/entrepreneur	13 (5.1)	19 (7.4)	0.492	0.416	0.218–1.112	0.088
Healthcare professionals	2 (0.8)	2 (0.8)	0.337	1.021	0.046–2.494	0.287
**Practices**						
Age (years)						
18–25	55 (21.4)	77 (30.0)	Ref.	–	–	–
26–40	37 (14.4)	20 (7.8)	0.338	0.340	0.173–0.657	0.001*
41–60	35 (13.6)	20 (7.8)	0.366	0.342	0.187–0.715	0.003*
>60	10 (3.9)	3 (1.2)	0.193	0.695	0.050–0.754	0.018*
Pet vaccination status						
Never received/no pets	55 (21.4)	30 (11.7)	Ref.	–	–	–
No pets requiring vaccination	10 (3.9)	6 (2.3)	1.167	0.584	0.372–3.664	0.792
Has received	72 (28.0)	84 (32.7)	2.512	0.294	1.413–4.465	0.002*

Ref = Reference group; AOR = Adjusted odds ratio; CI = Confidence interval; SE = Standard error. * Indicates statistical significance at p < 0.05.

### Association among KAP

Associations among KAP were evaluated using Pearson's chi-square test, and effect sizes were estimated using Phi coefficients (Table 3). Knowledge was significantly associated with attitudes (φ = 0.257, p = 0.001) and preventive practices (φ = 0.319, p < 0.001), indicating small-to-moderate positive associations. In contrast, attitudes and preventive practices showed no statistically significant association (φ = 0.121, p = 0.121).

**Table 3:** Association among KAP regarding rabies among participants.

**Association**	**Statistical test**	**Phi coefficient (Cramer's V)**	**p-value**
Knowledge vs. attitudes	Pearson's χ²	0.257	0.001*
Knowledge vs. practices	Pearson's χ²	0.319	<0.001*
Attitudes vs. practices	Pearson's χ²	0.121	0.121

* Indicates statistical significance at p < 0.05. CI = Confidence interval; KAP = Knowledge, attitudes, and practices. Associations among dichotomized KAP variables were evaluated using Pearson's chi-square test. Phi coefficients (equivalent to Cramer's V for 2 × 2 contingency tables) were used to estimate effect sizes.

## DISCUSSION

### Sociodemographic characteristics and study context

This study evaluated the KAP regarding rabies among residents from multiple districts of Bangkok, Thailand. The findings provide contemporary evidence regarding community awareness, perceptions, and preventive behaviors related to this important zoonotic disease in a highly urbanized setting. The high questionnaire completion rate (98.47%; 258/262) indicates excellent participant engagement and strengthens the reliability of the study findings. The demographic profile, characterized predominantly by young, well-educated female participants, is consistent with the population typically represented in online community surveys conducted in large metropolitan areas such as Bangkok.

An important strength of this study is that it examined sociodemographic determinants of KAP alongside the relationship between pet vaccination history and preventive practices. These findings support urban rabies prevention strategies and contribute to Thailand's ongoing efforts to achieve the WHO "Zero by 30" goal of eliminating dog-mediated human rabies.

### Public health implications and policy relevance

From a public health perspective, the findings provide important evidence for designing more effective rabies prevention strategies in urban settings. Although participants generally showed good awareness of rabies transmission and the importance of vaccination, knowledge gaps and differences across occupational and age groups suggest that existing health communication strategies may not reach all population segments [32, 33]. Public health authorities should therefore consider targeted educational interventions for groups with lower knowledge and preventive practices, particularly working adults and older individuals [34].

Integrating rabies education into workplace health programs, community outreach activities, and school-based health promotion initiatives may improve public awareness and reinforce preventive behaviors [35]. Furthermore, strengthening collaboration among veterinary services, healthcare providers, and local government authorities would facilitate more coordinated rabies surveillance, vaccination campaigns, and community education in accordance with the One Health approach [13].

For the Bangkok Metropolitan Administration, these findings support expanding workplace-based rabies education, strengthening community awareness campaigns, promoting responsible pet vaccination through local veterinary networks, and further integrating rabies surveillance into municipal One Health programs. Collectively, these targeted interventions could strengthen collaboration between veterinary and public health sectors and further support Thailand's progress toward eliminating dog-mediated human rabies by 2030.

### Knowledge of rabies and remaining knowledge gaps

The mean knowledge score of 7.75, with 58.9% of participants demonstrating sufficient knowledge, indicates a moderately well-informed urban population. This finding aligns with previous community-based KAP studies in Thailand, Cambodia, and the Philippines, which similarly reported strong awareness of rabies transmission and the importance of vaccination while identifying persistent gaps in post-exposure management and other preventive measures [24, 36, 37].

Participants showed particularly high knowledge of rabies transmission, clinical signs in dogs, and animal vaccination, suggesting that public health messaging has effectively communicated the fundamentals of rabies prevention. These findings are encouraging because understanding transmission routes and the protective role of vaccination is essential for reducing disease risk and promoting timely preventive actions [24].

Nevertheless, the study identified an important knowledge gap regarding the recommended observation period after a bite incident, with only 36.1% of participants providing the correct response. This deficiency has important public health implications because misunderstanding the appropriate observation period may result in inappropriate management of biting animals, delayed reporting, unnecessary post-exposure prophylaxis, or failure to seek appropriate medical advice [38]. Similar knowledge gaps in post-bite animal management have been reported in other countries [39, 40]. Addressing this specific knowledge gap should therefore be prioritized in future public education campaigns because appropriate management immediately after potential rabies exposure remains a cornerstone of effective rabies prevention [41].

### Attitudes toward rabies prevention and control

Participants generally demonstrated favorable attitudes toward rabies prevention, as reflected by the mean attitude score of 7.72. Nearly all respondents recognized the importance of vaccinating dogs against rabies, highlighting broad public acceptance of one of the most effective rabies control measures. This observation is consistent with previous studies emphasizing the central role of canine vaccination in reducing rabies transmission [36].

In contrast, attitudes toward the humane euthanasia of laboratory-confirmed rabid dogs and participants' confidence in recognizing rabies clinical signs were considerably less favorable. These findings suggest that cultural beliefs, ethical considerations, and limited confidence in recognizing rabies may influence decision-making related to rabies prevention and control. Similar variations in public perceptions regarding euthanasia have been reported in other rabies endemic settings [42–44].

In Thailand, compassion toward animals is strongly influenced by cultural and religious values, which may contribute to lower public acceptance of euthanasia despite its importance in preventing further rabies transmission. Consequently, educational programs should clearly communicate the public health rationale for the humane euthanasia of laboratory-confirmed rabid animals while remaining sensitive to local cultural and ethical perspectives. Improving public recognition of rabies clinical signs should also be emphasized because early identification of suspected rabid animals facilitates timely reporting and implementation of appropriate control measures [45].

### Rabies-related practices and behavioral patterns

The mean practice score of 11.05 indicates generally appropriate rabies prevention practices among participants. Most respondents reported routine pet rabies vaccination, avoiding aggressive stray dogs, washing wounds immediately after suspected rabies exposure, and seeking prompt medical consultation after animal bites. These behaviors are consistent with internationally recommended rabies prevention measures and represent important protective practices for reducing the risk of human rabies [45].

Despite these encouraging findings, participation in rabies-related education and awareness activities remained limited, with only 26.7% of respondents reporting previous involvement in such programs. This finding suggests that although participants generally practiced appropriate preventive behaviors, active engagement in community-based rabies education remains suboptimal [46]. Continuous public education has consistently been identified as an essential component of sustainable rabies prevention programs [24].

Similarly, only 60.5% of participants reported having received rabies vaccination themselves. Because pre-exposure vaccination is generally recommended only for individuals at increased occupational or exposure risk, this finding should be interpreted cautiously and may not necessarily indicate inadequate preventive behavior. Instead, it may reflect the relatively small proportion of participants in high-risk groups or limited awareness of pre-exposure vaccination recommendations. Previous studies in Southeast Asia have identified limited vaccine accessibility, financial constraints, insufficient public awareness, and inadequate intersectoral coordination as continuing barriers to rabies prevention [15, 45–47].

Determinants of KAP: Sociodemographic influences

The present study identified several sociodemographic factors associated with rabies-related KAP. Multivariable logistic regression showed that occupation independently predicted both knowledge and attitudes, whereas age and pet vaccination history were independently associated with preventive practices. These findings indicate that demographic characteristics influence rabies awareness and preventive behaviors among urban residents and may help identify population groups requiring targeted public health interventions.

For rabies knowledge, occupation remained a significant predictor after adjustment for potential confounders. Compared with students or unemployed participants, private sector employees had significantly lower odds of demonstrating sufficient knowledge, and self-employed participants also showed reduced odds, although the association was borderline statistically significant. Although the healthcare professional subgroup also demonstrated variability, the corresponding AOR estimates should be interpreted cautiously because only four participants were included in this category, resulting in wide CIs and reduced estimate precision. Nevertheless, the overall pattern observed among the major occupational groups remained consistent with the study findings. These results suggest that access to rabies information may differ according to occupation, possibly reflecting variations in exposure to public health education, workplace health promotion, or opportunities to participate in awareness activities. Previous studies have reported similar associations between socioeconomic characteristics and zoonotic disease awareness [24, 34, 41, 48, 49].

Occupation also remained independently associated with attitudes toward rabies prevention. Participants employed in the private sector were less likely to demonstrate positive attitudes than students or unemployed participants. These findings suggest that workplace commitments and limited participation in health promotion activities may influence perceptions of rabies prevention. Tailored educational interventions targeting occupational groups with lower awareness may therefore improve both knowledge and attitudes toward rabies prevention.

Regarding preventive practices, age and pet vaccination history were identified as independent predictors. Participants aged >25 years were significantly less likely to report appropriate preventive practices than those aged 18-25 years, whereas participants whose pets had received rabies vaccination were more than twice as likely to demonstrate appropriate preventive behaviors. These findings emphasize the importance of responsible pet ownership in promoting broader community engagement in rabies prevention. A previous study has reported similar associations between responsible pet ownership and preventive behaviors [50].

The better preventive practices observed among younger participants may reflect greater exposure to digital health information, social media campaigns, and university-based health education, which have become major channels for health communication in Thailand. Younger adults may also seek health information online more frequently than older individuals. Likewise, the lower knowledge and attitude scores among private sector employees may relate to demanding work schedules and fewer opportunities to participate in community-based health education activities. These findings suggest that workplace-based rabies awareness programs could effectively complement existing community education initiatives. Furthermore, characteristics of urban living in Bangkok, including intensive work schedules, reliance on digital communication, and frequent interactions among people, companion animals, and free-roaming animals, may further influence access to rabies education and adoption of preventive behaviors. However, because of the cross-sectional design, these findings should be interpreted as associations rather than causal relationships.

### Implications for targeted interventions

An important implication of this study is the identification of demographic groups that may benefit from targeted rabies education. The associations between occupation and both knowledge and attitudes suggest that access to rabies information differs across occupational groups, potentially because of variations in exposure to public health messaging or participation in educational activities. Similarly, the lower likelihood of appropriate preventive practices among older participants may reflect age-related differences in health literacy, access to digital information, or risk perception. The positive association between pet vaccination history and preventive practices further highlights the importance of promoting responsible pet ownership as part of comprehensive community-based rabies prevention strategies. These findings support tailored educational interventions targeting specific demographic groups rather than relying solely on generalized public awareness campaigns.

### Interrelationship among KAP

A principal finding of this study was the significant positive association between knowledge and both attitudes and preventive practices. Participants with higher knowledge levels were more likely to demonstrate favorable attitudes toward rabies prevention and appropriate preventive behaviors. These findings align with the KAP framework, which proposes that knowledge underpins positive attitudes and promotes appropriate health behaviors [48].

In contrast, no significant association was observed between attitudes and preventive practices. This finding suggests that favorable attitudes alone may not translate into appropriate preventive behaviors. Practical barriers, perceived risk, access to veterinary and healthcare services, and opportunities to participate in health education may influence behavior adoption independently of attitudes. Consequently, interventions to improve rabies prevention should focus not only on promoting positive attitudes but also on strengthening knowledge and addressing barriers that may prevent appropriate behavioral change. Nevertheless, the cross-sectional design prevents causal inference.

### One Health perspective in rabies control

Rabies prevention represents a classical application of the One Health concept, recognizing the close interdependence of human, animal, and environmental health [51]. Effective rabies control requires coordinated collaboration among veterinary services, public health authorities, local governments, and communities to simultaneously reduce human exposure and control infection in animal reservoirs [15].

The present findings reinforce the importance of integrating community education with animal vaccination programs, responsible pet ownership, and management of free-roaming dog populations [52]. In Bangkok, where high population density, widespread pet ownership, and frequent interactions with free-roaming animals create favorable conditions for rabies transmission, coordinated urban rabies control is particularly important. Public education campaigns, accessible veterinary services, integrated surveillance systems, and responsible pet ownership should therefore be implemented through close collaboration among municipal authorities, veterinary services, and healthcare providers. The observed association between pet vaccination history and appropriate preventive practices further supports integrated One Health approaches that simultaneously promote animal vaccination, responsible pet ownership, and human rabies prevention. Strengthening these integrated One Health strategies will contribute to Thailand's national rabies elimination program and support achievement of the WHO "Zero by 30" initiative.

### Strengths and limitations

This study has several important strengths. It provides one of the most recent assessments of rabies-related KAP among adults residing in metropolitan Bangkok, a highly urbanized environment with distinctive challenges for rabies prevention and control. The questionnaire underwent structured validation with expert review, and participants were recruited from multiple districts across Bangkok, providing broad geographic representation within the study area. Multivariable logistic regression also identified independent predictors of KAP while adjusting for potential confounders. Identifying occupation as a determinant of rabies knowledge and attitudes, along with the positive association between pet vaccination history and preventive practices, provides contemporary evidence that may help develop targeted urban One Health interventions.

This study also has several limitations. First, the cross-sectional design precludes establishing causal relationships between participant characteristics and KAP outcomes; therefore, the observed associations should be interpreted cautiously. Second, participants were recruited through non-probability convenience sampling via online platforms, which may have introduced selection bias by preferentially recruiting younger, well-educated, and digitally connected individuals. Consequently, the findings may not be fully generalizable to the broader population of Bangkok or other regions of Thailand. Third, self-reported responses are subject to recall and social desirability bias, potentially overestimating appropriate knowledge and preventive behaviors. Fourth, although multivariable analyses adjusted for measured covariates, residual confounding from unmeasured factors, including prior exposure to rabies education, access to veterinary services, and socioeconomic status, cannot be ruled out. In addition, the healthcare professional subgroup comprised only four participants, resulting in wide CIs and limited precision of the corresponding AOR estimates. Finally, dichotomizing attitude and practice scores, although widely adopted in KAP studies, may have reduced response variability and resulted in some loss of information compared with analyses based on the original Likert scale responses. Despite these limitations, the study provides valuable baseline evidence regarding rabies-related KAP among urban residents in Bangkok and offers useful information for developing targeted public health interventions.

### Future directions

Future studies should build on these findings by conducting longitudinal investigations to evaluate changes in rabies-related KAP following educational interventions and public awareness campaigns. Mixed methods studies incorporating qualitative interviews would provide deeper insight into behavioral barriers, cultural influences, and perceptions affecting rabies prevention. In addition, intervention studies evaluating workplace-based education, school-based awareness programs, community engagement initiatives, and responsible pet ownership interventions would generate valuable evidence for optimizing urban rabies prevention strategies. Further research involving more representative population-based sampling from diverse regions of Thailand would also improve the generalizability of findings and strengthen national rabies control policies in support of the WHO "Zero by 30" initiative.

## CONCLUSION

This study showed that Bangkok residents generally had favorable rabies-related knowledge, attitudes, and preventive practices, although important gaps remained in specific aspects of rabies prevention. Overall, 58.9% of participants demonstrated sufficient knowledge, with particularly high awareness of rabies transmission, clinical signs in dogs, and the importance of animal vaccination. However, knowledge regarding the recommended observation period following a bite incident remained limited. Participants also exhibited generally positive attitudes toward rabies prevention, particularly regarding the importance of dog vaccination, whereas attitudes toward the humane euthanasia of laboratory-confirmed rabid animals and confidence in recognizing rabies signs were less favorable. Preventive practices were generally appropriate, including routine pet vaccination, immediate wound washing, and prompt medical consultation after suspected rabies exposure, although participation in rabies awareness programs remained relatively low.

Multivariable analysis identified occupation as an independent determinant of rabies knowledge and attitudes, while age and pet vaccination history were independently associated with preventive practices. Furthermore, higher knowledge levels were positively associated with both favorable attitudes and appropriate preventive behaviors, emphasizing the central role of knowledge in promoting effective rabies prevention.

The findings have important practical implications for urban rabies control. Targeted educational interventions should prioritize occupational groups with lower awareness, older adults, and individuals with limited participation in rabies education programs. Integrating workplace-based education, community outreach, responsible pet ownership, and routine pet vaccination into municipal One Health initiatives may strengthen public engagement, improve preventive behaviors, and support coordinated rabies surveillance and control. Such evidence-based strategies are particularly relevant for Bangkok and other rapidly urbanizing cities facing similar rabies prevention challenges.

In conclusion, strengthening public knowledge through targeted education, promoting responsible pet ownership and vaccination, and reinforcing collaboration among veterinary, public health, and municipal sectors are essential to improving rabies prevention in urban communities. These integrated strategies will support sustainable rabies control in Thailand and progress toward the WHO "Zero by 30" goal of eliminating dog-mediated human rabies.

## DATA AVAILABILITY

The supplementary data are available from the corresponding author upon request.

## GENERATIVE AI DECLARATION

The authors declare that generative artificial intelligence (AI) tools were used solely to improve language, grammar, and readability during manuscript preparation. All scientific content, data analysis, interpretation of results, and conclusions were developed and verified by the authors. The authors take full responsibility for the accuracy, integrity, and originality of the work presented, and no AI tool was listed as an author.

## AUTHOR’S CONTRIBUTIONS

KL, TB, RL, SC, AP, SVM, MTK and AT: Conceptualization, study design, data analysis, and writing the original draft. SJ: Conceptualization, study design, data analysis, supervision, writing the original draft, and manuscript review and editing. All authors have read and approved the final manuscript.

## References

[1] Farahtaj F, Alizadeh L, Gholami A, Tahamtan A, Shirian S, Fazeli M (2019). Natural infection with rabies virus: a histopathological and immunohistochemical study of human brains. Osong Public Health Res Perspect.

[2] Tkachev S Introductory Chapter: Rabies in the 21st Century. Rabies Virus at the Beginning of 21st Century. 2022;3.

[3] Hopkins J, Sweck S, S R, Tkachev S The Diagnosis, Clinical Course, Treatment, and Prevention of the Rabies Virus. Rabies Virus at the Beginning of 21st Century. IntechOpen; 2022. p. 19.

[4] Wu J, Qi Y, Zhang W, Liu L, Chen J, Yang Y (2025). Metagenomic next-generation sequencing for etiological diagnosis of an unexpected rabies case with unclear exposure history. BMC Infect Dis.

[5] Minoungou G, Dahourou LD, Savadogo M, Tialla D, Combari AHB, Kanyala E (2021). Surveillance of animal rabies in Burkina Faso: a retrospective laboratory data from 2008 to 2012. Int J Vet Sci.

[6] Pal M, Zende R, Bekele A, Rebuma T, Panicker N (2025). A knowledge update on current strategies on the prevention and control of rabies in Ethiopia. Indian J Anim Health.

[7] Guo X, Zhang M, Feng Y, Liu X, Wang C, Zhang Y (2024). Transcriptome analysis of salivary glands of rabies-virus-infected mice. Front Microbiol.

[8] Potratz M, Zaeck LM, Weigel C, Klein A, Freuling CM, Müller T (2020). Neuroglia infection by rabies virus after anterograde virus spread in peripheral neurons. Acta Neuropathol Commun.

[9] Gossner CM, Mailles A, Aznar I, Dimina E, Echevarría JE, Feruglio SL (2020). Prevention of human rabies: a challenge for the European Union and the European Economic Area. Euro Surveill.

[10] Yang C, Dang M, Wu L, Jin G, Deng Q, Chen Y (2025). Disparities in Global Rabies Burden from GBD 2021: Children, older adults, and low-SDI countries at continued risk. PLoS Negl Trop Dis.

[11] Punyapornwithaya V, Thanapongtharm W, Jainonthee C, Chinsorn P, Sagarasaeranee O, Salvador R (2023). Time series analysis and forecasting of the number of canine rabies confirmed cases in Thailand based on national-level surveillance data. Front Vet Sci.

[12] Thanapongtharm W, Suwanpakdee S, Chumkaeo A, Gilbert M, Wiratsudakul A (2021). Current characteristics of animal rabies cases in Thailand and relevant risk factors identified by a spatial modeling approach. PLoS Negl Trop Dis.

[13] Kiratitana-Olan K, Chaisowwong W, Thongkorn K, Kreausukon K (2022). One Health perspectives on sustainable rabies prevention in Thailand: a qualitative interview study. Vet Integr Sci.

[14] Lyabangi JF, Mahulu EM, Sandetwa J, John WA, Madoshi PB (2025). Exploring Global Strategies to Combat Rabies: Addressing Limitations and Enhancing Effectiveness. Vet Med Sci.

[15] Tiwari HK, Gogoi-Tiwari J, Robertson ID (2021). Eliminating dog-mediated rabies: challenges and strategies. Anim Dis.

[16] Vega S, Lorenzo-Rebenaque L, Marin C, Domingo R, Fariñas F (2021). Tackling the threat of rabies reintroduction in Europe. Front Vet Sci.

[17] Jacobsen KH (2020).

[18] Andrade C, Menon V, Ameen S, Kumar Praharaj S (2020). Designing and conducting knowledge, attitude, and practice surveys in psychiatry: practical guidance. Indian J Psychol Med.

[19] Porsuk AO, Cerit C (2021). An increasing public health problem: Suspected rabies exposures. The Journal of Infection in Developing Countries.

[20] Akankwatsa D, Bagonza A, Kiguli J, Kambugu A, Lamorde M, Okech SG (2025). Barriers to rabies control through mass dog vaccination in rural Uganda: Insights from community perspectives and key informant interviews. PLoS Negl Trop Dis.

[21] Swedberg C, Bote K, Gamble L, Fénelon N, King A, Wallace RM (2024). Eliminating invisible deaths: the woeful state of global rabies data and its impact on progress towards 2030 sustainable development goals for neglected tropical diseases. Front Trop Dis.

[22] Laorujisawat M, Wattanaburanon A, Abdullakasim P, Maharatchpong N (2022). Rabies-related knowledge, attitudes, and practices among primary school students in Chonburi Province, Thailand. INQUIRY: The Journal of Health Care Organization, Provision, and Financing.

[23] Thongyuan S, Pinyopummintr T, Sithisarn P, Sinthusing C, Sagarasaeranee O, Thichumpa W (2016). Knowledge and practices regarding rabies in urban and rural communities in Thailand and Cambodia. Int J Infect Dis.

[24] Premashthira S, Suwanpakdee S, Thanapongtharm W, Sagarasaeranee O, Thichumpa W, Sararat C (2021). The impact of socioeconomic factors on knowledge, attitudes, and practices of dog owners on dog rabies control in Thailand. Front Vet Sci.

[25] Hiby E, Rungpatana T, Izydorczyk A, Benka V, Rooney C (2025). Population demographics of owned dogs in Greater Bangkok and implications for free-roaming dog population management. Animals.

[26] Leelakajornkit S, Dumniem N, Wataganara P, Maison S, Ponglowhapan S (2024). Impacts of Species, Gender Disparities, and COVID-19 Lockdowns on Population Control: Insights from 337,590 Dogs and Cats in Bangkok Metropolitan Administration (BMA), Thailand (2009-2023). Thai J Vet Med.

[27] Chavanayarn A (2024).

[28] Sergeant E Epitools epidemiological calculators. Ausvet Bruce; 2018. [Cited Sept 15, 2026]. Available from: http://epitools.ausvet.com.au.

[29] Srimoragot P, Sudsakorn P, Suwannaphirom P, Ruchisereekul K (2021). Survey of Knowledge and Opinion among Thai Veterinarians on Nicotine Toxicity and Second-hand Smoke Effects on Pets’ Health+. Thai J Vet Med.

[30] Salah M, Zaitoun Y, Fakhry B, Karayni M, Mokashar A, Chahine C (2024). Knowledge, attitude, and practices concerning prostate cancer and its prevention in the Lebanese population. Sci Rep.

[31] Masoud AT, Zaazouee MS, Elsayed SM, Ragab KM, Kamal EM, Alnasser YT (2021). KAP-COVIDGLOBAL: a multinational survey of the levels and determinants of public knowledge, attitudes and practices towards COVID-19. BMJ Open.

[32] Krishnamoorthy Y, Vijayageetha M, Sarkar S (2018). Awareness about rabies among general population and treatment seeking behaviour following dog bite in rural Puducherry: A community-based cross-sectional study. Int J Community Med Public Health.

[33] Shaikh SU, Zaidi F, Shaikh Z, Fahim MF (2022). Rabies: Perils and prevention, an insight into practices and perception in residents of Pakistan. Iran Biomed J.

[34] Pal P, Yawongsa A, Bhusal TN, Bashyal R, Rukkwamsuk T (2021). Knowledge, attitude, and practice about rabies prevention and control: A community survey in Nepal. Vet World.

[35] Shaikh A, Habib SS, Saleem AF, Salahuddin N (2025). Towards Rabies Elimination in Pakistan: Barriers, Facilitators, and the Role of One Health. J Epidemiol Glob Health.

[36] Ung B, Kamyingkird K, Phimpraphai W (2021). Knowledge, attitude, and practices associated with rabies in villages with different dog vaccination statuses in Cambodia. Vet World.

[37] Lañada EB, Quilicot AMM, Balala LM, Cortez ES, Peña Jr ST, Torregoza RB (2019). Knowledge, attitudes, and practices relating to rabies control in Baybay City, Leyte, Philippines. Ann Trop Res.

[38] Penjor K, Tenzin T, Jamtsho RK (2019). Determinants of health seeking behavior of animal bite victims in rabies endemic South Bhutan: a community-based contact-tracing survey. BMC Public Health.

[39] Kabeta T, Deresa B, Tigre W, Ward MP, Mor SM (2015). Knowledge, attitudes and practices of animal bite victims attending an anti-rabies health center in Jimma Town, Ethiopia. PLoS Negl Trop Dis.

[40] Liu Q, Wang X, Liu B, Gong Y, Mkandawire N, Li W (2017). Improper wound treatment and delay of rabies post-exposure prophylaxis of animal bite victims in China: Prevalence and determinants. PLoS Negl Trop Dis.

[41] Leblanc C, Kassié D, Ranaivoharimina M, Rakotomanana EFN, Mangahasimbola RT, Randrianarijaona A (2024). Mixed methods to evaluate knowledge, attitudes and practices (KAP) towards rabies in central and remote communities of Moramanga district, Madagascar. PLoS Negl Trop Dis.

[42] Martinez E, Cesário CS, Dias JV, Silva IO, Souza VB (2018). Community perception and attitudes about the behavior of stray dogs in a college campus. Acta Vet Bras.

[43] Widyastuti MDW, Bardosh KL, Basri C, Basuno E, Jatikusumah A, Sunandar (2015). On dogs, people, and a rabies epidemic: results from a sociocultural study in Bali, Indonesia. Infect Dis Poverty.

[44] Nadal D, Hampson K, Lembo T, Rodrigues R, Vanak AT, Cleaveland S (2022). Where rabies is not a disease. Bridging healthworlds to improve mutual understanding and prevention of rabies. Front Vet Sci.

[45] Amparo ACB, Jayme SI, Roces MCR, Quizon MCL, Mercado MLL, Dela Cruz MPZ (2018). The evaluation of Animal Bite Treatment Centers in the Philippines from a patient perspective. PLoS One.

[46] Ling MYJ, Halim AFNA, Ahmad D, Ramly N, Hassan MR, Rahim SSSA (2023). Rabies in Southeast Asia: a systematic review of its incidence, risk factors and mortality. BMJ Open.

[47] Slack V, Nadal D, Yasobant S, Cliquet F, Ahmad W, Pushpakumara N (2023).

[48] Ahmed MJ, Bhuiyan MIH, Chalise R, Mamun M, Bhandari P, Islam K (2025). One health assessment of farmers’ knowledge, attitudes, and practices (KAPs) on zoonoses in Bangladesh. Sci Rep.

[49] Ahmed T, Hussain S, Zia U-u-R, Rinchen S, Yasir A, Ahmed S (2020). Knowledge, attitude and practice (KAP) survey of canine rabies in Khyber Pakhtunkhwa and Punjab Province of Pakistan. BMC Public Health.

[50] Lañada EDA, Glova NM, Lañada EB (2023). Predicting preventive behaviors on Dog-Borne Zoonoses using the Health Belief Model. Ann Trop Res.

[51] Fahrion AS, Freuling CM, Léchenne M, Müller T, Recuenco S, Vigilato MA (2023). Rabies, a long-standing One Health example–progress, challenges, lessons and visions on the way to 0 by 30. Front Vet Sci.

[52] Marjiana T, Astari AM, Zuhriyah L (2021). Systematic review of community efforts in early handling post-exposure prophylaxis cases of rabies animal bite transmission. Int J Public Health.

